# Characterizations of the multi-kingdom gut microbiota in Chinese patients with gouty arthritis

**DOI:** 10.1186/s12866-023-03097-0

**Published:** 2023-11-24

**Authors:** Changming Chen, Yue Zhang, Xueming Yao, Qiulong Yan, Shenghui Li, Qin Zhong, Zhengqi Liu, Fang Tang, Can Liu, Hufan Li, Dan Zhu, Weiya Lan, Yi Ling, Daomin Lu, Hui Xu, Qiaoyi Ning, Ying Wang, Zong Jiang, Qiongyu Zhang, Guangzhao Gu, Liping Sun, Nan Wang, Guangyang Wang, Aiqin Zhang, Hayan Ullah, Wen Sun, Wukai Ma

**Affiliations:** 1 Department of Rheumatology and Immunology, The Second Affiliated Hospital of Guizhou University of Traditional Chinese Medicine, Guiyang, China; 2 Puensum Genetech Institute, Wuhan, China; 3 Department of Microbiology, College of Basic Medical Sciences, Dalian Medical University, Dalian, China; 4 Key Laboratory of Health Cultivation of the Ministry of Education, Beijing University of Chinese Medicine, Beijing, China

**Keywords:** Gouty arthritis, Whole-metagenome shotgun sequencing, Gut bacteriome, Gut mycobiome, Gut virome, Microbiota dysbiosis, Multi-kingdom signatures

## Abstract

**Objective:**

The gut microbial composition has been linked to metabolic and autoimmune diseases, including arthritis. However, there is a dearth of knowledge on the gut bacteriome, mycobiome, and virome in patients with gouty arthritis (GA).

**Methods:**

We conducted a comprehensive analysis of the multi-kingdom gut microbiome of 26 GA patients and 28 healthy controls, using whole-metagenome shotgun sequencing of their stool samples.

**Results:**

Profound alterations were observed in the gut bacteriome, mycobiome, and virome of GA patients. We identified 1,117 differentially abundant bacterial species, 23 fungal species, and 4,115 viral operational taxonomic units (vOTUs). GA-enriched bacteria included *Escherichia coli_D* GENOME144544, *Bifidobacterium infantis* GENOME095938, *Blautia_A wexlerae* GENOME096067, and *Klebsiella pneumoniae* GENOME147598, while control-enriched bacteria comprised *Faecalibacterium prausnitzii_G* GENOME147678, *Agathobacter rectalis* GENOME143712, and *Bacteroides_A plebeius_A* GENOME239725. GA-enriched fungi included opportunistic pathogens like *Cryptococcus neoformans* GCA_011057565, *Candida parapsilosis* GCA_000182765, and *Malassezia* spp., while control-enriched fungi featured several *Hortaea werneckii* subclades and *Aspergillus fumigatus* GCA_000002655. GA-enriched vOTUs mainly attributed to *Siphoviridae*, *Myoviridae*, *Podoviridae*, and *Microviridae*, whereas control-enriched vOTUs spanned 13 families, including *Siphoviridae*, *Myoviridae*, *Podoviridae*, *Quimbyviridae*, *Phycodnaviridae*, and *crAss-like*. A co-abundance network revealed intricate interactions among these multi-kingdom signatures, signifying their collective influence on the disease. Furthermore, these microbial signatures demonstrated the potential to effectively discriminate between patients and controls, highlighting their diagnostic utility.

**Conclusions:**

This study yields crucial insights into the characteristics of the GA microbiota that may inform future mechanistic and therapeutic investigations.

**Supplementary Information:**

The online version contains supplementary material available at 10.1186/s12866-023-03097-0.

## Key messages

(1) Gut bacteriome, mycobiome, and virome of GA patients were substantially altered compared with controls.

(2) GA-enriched bacteria and fungi include potential pathogens.

(3) Multi-kingdom microbial signatures may be used to predict and discriminate GA patients and controls.

**Supplementary Information:**

The online version contains supplementary material available at 10.1186/s12866-023-03097-0.

## Introduction

Gouty arthritis (GA) is a multifaceted disease characterized by prolonged purine metabolism disorder and elevated blood uric acid levels, resulting in tissue and organ damage [1]. GA arises from disorders in purine metabolism or reduced uric acid excretion, which leads to the deposition of monosodium urate crystals within or around joints. Clinically, this manifested as evident redness, swelling, heat, and pain in the soft tissue of joints [2]. Typically, the first metatarsophalangeal joint is affected, but larger joints can also be involved, giving rise to systemic acute inflammation [3]. GA stands as one of the most prevalent inflammatory arthritic conditions, with a global prevalence of approximately 2–4%, primarily affecting men over 40 [4]. It is often accompanied by comorbidities such as obesity, coronary artery disease, hypertension, metabolic disease, or diabetes.

Accumulating evidence suggests that the gut microbiota plays a pivotal role in various human diseases. Autoimmune or metabolic conditions, including rheumatoid arthritis (RA) [5], osteoarthritis (OA) [6], inflammatory bowel disease (IBD) [7], systemic lupus erythematosus (SLE) [8, 9], and diabetes [10], have all been linked to alterations in the gut microbiota. Dysbiosis of the gut microbiota can affect intestinal epithelium permeability, disturb immune tolerance, and activate immune cells, ultimately resulting in joint inflammation and bone destruction in RA patients [5]. Compared with healthy subjects, gut microbial diversity in RA patients was significantly reduced [11]. *Prevotella*, more prevalent in the early stages of RA, has been implicated in promoting RA pathogenesis through its mediation of Th17 cell inflammatory responses [12]. Similarly, gut microbiota dysbiosis in SLE patients exhibits proinflammatory and autoimmune features [13]. Furthermore, SLE patients tend to exhibit reduced richness and diversity in their gut microbiota, especially those with higher SLEDAI scores [14]. These findings underscore the potential involvement of the gut microbiota in immune diseases, perhaps even as an inducing factor. Additionally, gut fungi and viruses are also related to immune diseases; for instance, the reduction of fungi clades *Pholiota*, *Scedosporium*, and *Trichosporon* is closely associated with RA [15]. Guo et al. reported that the perturbations of the viral compositions of gut and oral and the networks associated with microbes may contribute to the pathogenesis of RA [16].

These observations suggest a potential influence of the gut microbiota on GA. Prior microbiota studies in GA, which were primarily based on 16S rRNA sequencing, reported increased *Enterobacteriaceae* during gout's acute state [17]. *Bacteroides* was found to be more enriched in GA patients, with *Escherichia* and *Shigella* from the *Enterobacteriaceae* family being more abundant in patients with tophi compared to the general population [18]. It is important to note that the gut microbiota plays an essential role in the activation of the NLRP3 inflammasome in GA [19]. Park et al. identified substantial shifts in bacterial composition and increased production of short-chain fatty acids (SCFAs) (especially acetate) after treating GA patients [20].

Nevertheless, these studies have predominantly focused on gut bacteria, lacking a comprehensive analysis of the gut mycobiome and virome in patients. In this study, we conducted an extensive profiling of the gut bacteriome, mycobiome, and virome in 26 GA patients and 28 healthy controls using deep whole-metagenome shotgun sequencing of their fecal samples. Our investigation scrutinized the associations among multi-kingdom signatures associated with GA, providing valuable insights into the role of gut microorganisms in GA development. These findings may offer translational prospects for the prevention and treatment of GA and related diseases.

## Methods

### Subject recruitment

Twenty-six gouty arthritis patients admitted to the Department of Rheumatology and Immunology, Second Affiliated Hospital of Guizhou University of Chinese Medicine, China, between August 2020 and August 2021, were recruited in this study. All the patients fulfilled the guidelines of the 2015 American College of Rheumatology/European League Against Rheumatism (ACR/EULAR) classification criteria [21]. Twenty-eight healthy subjects were recruited from the same hospital based on the previously described methods [6]. The study had exclusion criteria for both GA patients and healthy controls, which included: (1) volunteers with other metabolic diseases, acute and chronic renal failure, digestive system diseases, tuberculosis, and opportunistic infections; (2) volunteers with excessive drinking habits, and all participants who had drinking sour milk within 1 week; (3) volunteers who received antibiotics, antifungals, or probiotics treatment in 1 month. The research protocol was approved by the Medical Ethical Committees of the Second Affiliated Hospital of Guizhou University of Traditional Chinese Medicine (approval number KY2023001 and KYW2023005). All subjects who participated in this study provided written informed consent in accordance with the Declaration of Helsinki.

Twenty-four out of 26 GA patients were female, which represented a slightly higher proportion than in the healthy control group (where 20 out of 28 were female), but this difference was not statistically significant (Fisher’s exact test *p* = 0.079). Furthermore, there were no significant differences in age (average 45.2 ± 8.8 for patients vs. 49.5 ± 8.7 for controls, Student’s t-test *p* = 0.603) and body mass index (BMI) (24.7 ± 3.4 vs. 23.2 ± 2.8, *p* = 0.091) between the patients and healthy individuals.

### Fecal sample collection and sequencing

Fecal samples of all participants were collected immediately following defecation and transferred onto dry ice, and the weight of each collected fecal sample was more than 5g. Subsequently, the samples were transported to the laboratory, where they were divided into two equal portions and stored in two separate frozen tubes. All fecal samples were preserved at a temperature of -80°C. DNA extraction and whole-metagenome shotgun sequencing were performed based on the methods described in our previous study [6]. Briefly, the total DNA content of each fecal sample, approximately 170 mg per sample, was extracted using the Tiangen fecal DNA extraction kit, following the manufacturer's instructions. The concentration and purity of the extracted DNA were assessed using NanoDrop2000 and Qubit 4.0. To facilitate further analysis, the total DNA underwent fragmentation utilizing the Covaris M220 instrument (Gene Company Limited, China). A 150-bp paired-end library with an insert size of around 350 bp was constructed for each DNA sample. These libraries were barcoded and combined into a single pool for whole-metagenome shotgun sequencing on the Illumina NovaSeq platform. The initial base calling of the metagenomic dataset adhered to the default parameters of the sequencing platform. Raw sequencing reads for each sample underwent independent processing using the fastp [22], which trimmed low-quality bases (Q < 30) from the end of reads and filtered out reads containing N, those contaminated with adapters, or those shorter than 90 bp. Human reads were removed from the high-quality reads by aligning them against the human reference genome (GRCh38) using Bowtie2 [23].

### Bioinformatic analyses

Metagenomic reads from all samples were aligned against the Unified Human Gastrointestinal Genome (UHGG) database [24] to generate the profiles of the gut bacteriome. Reads that mapped to bacterial rRNA/tRNA gene sequences were excluded. The relative abundances at the phylum and genus levels were obtained by summing the abundances of species belonging to the same taxa. The functional composition of the fecal metagenomes was determined through the use of the HUMAnN3 algorithm [25].

To profile the gut mycobiome composition in fecal samples, we downloaded fungal genomes available from the National Center for Biotechnology Information (NCBI) RefSeq database. Specifically, we included fungal strains isolated from or found in human feces and digestive tract specimens, totaling 1,503 fungi. Metagenome reads from each sample were then aligned with the fungal genome references to construct the gut fungal profiles. Reads that mapped to the fungal rRNA/tRNA gene sequences were removed. To mitigate potential contamination from other gut microbes (e.g., bacteria, archaea, and viruses), reads aligned with fungal genomes underwent further alignment against (i) all bacterial, archaeal, or viral sequences extracted from the NCBI NT database and (ii) prokaryotic genomes from the UHGG database. Any contaminating reads thus identified were eliminated. The relative abundances of fungal species were normalized for each sample, and the relative abundances at the family and genus levels were determined by summing the species within the same taxa.

We employed metagenomic reads for de novo assembly using MEGAHIT [26], identifying viral sequences from the assembled contigs with a minimum length of 5,000 bp, following established methodologies [27–30]. The quality of viruses was assessed using CheckV [31]. The identified viral contigs were dereplicated at 95% sequence similarity and over 70% coverage to generate viral operational taxonomic units (vOTUs). Taxonomic annotation of vOTUs was performed through a method combining the GenomeNet Virus-Host Database [32] and the vConTACT2 pipeline [33]. To identify potential bacterial hosts for viruses, the CRISPR spacers of bacterial genomes were compared in a BLAST search against viral sequences with a bit score exceeding 50. Functional annotation of vOTUs was conducted based on the KEGG database [34].

A correlation analysis was performed among bacterial species, fungal species, and vOTUs using Spearman's rank correlation coefficient. Only correlation coefficients greater than 0.6 (positive) or less than -0.6 (negative) were considered to indicate strong correlations. The correlation network was visualized using Cytoscape [35].

### Statistical analyses

Statistical analyses were conducted using the R platform. To assess taxonomic and functional composition diversity, Shannon and Simpson diversity indices were calculated from the relative abundance profiles using the *vegan* package (version: 2.6–4). Principal coordinate analysis (PCoA) of Bray–Curtis distances was performed employing the *vegan* package. The dissimilarity in community composition was evaluated with permutational multivariate analysis of variance (PERMANOVA) using the *adonis* function from the *vegan* package, and the corresponding *adonis p*-value was generated through 1,000 permutations. For comparisons between two cohorts, Student's t-test and the Wilcoxon rank-sum test were employed to measure statistical differences. To account for multiple testing, the Benjamini–Hochberg procedure was applied, generating *q*-values. Statistical significance was set at a *p*-value (for individual tests) or a q-value (for multiple testing) of less than 0.05. In order to distinguish between GA patients and healthy controls using the abundance profiles of differentially abundant bacteria, fungi, and vOTUs, random forest models were executed with the *randomForest* package, involving the creation of 1,000 trees for classification.

## Results

### Diversity, phylogenetic and functional comparisons of the gut bacteriome

**Fig. 1:**
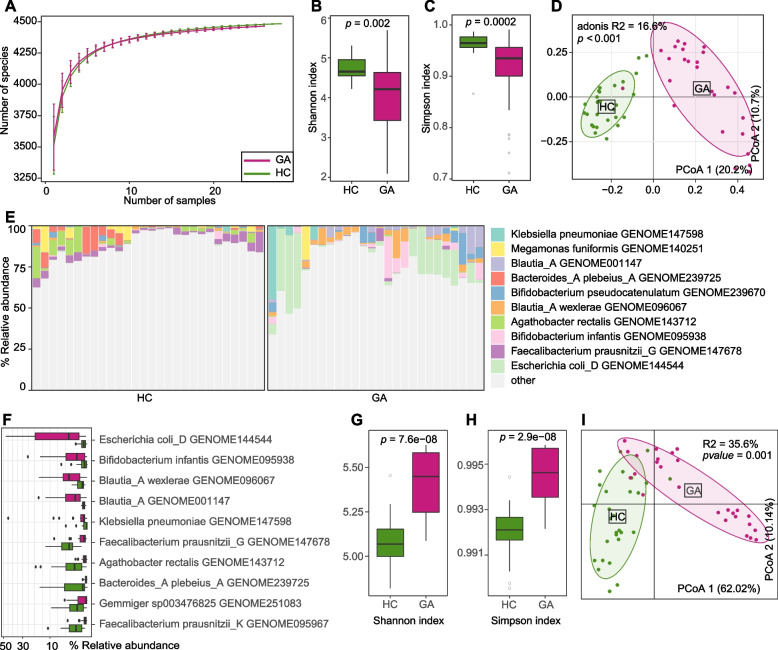
Difference in the gut bacteriome between GA patients and healthy controls. **A**, Rarefaction curve analysis of the number of observed species in two groups. The number of species in different groups is calculated based on a randomly selected specific number of samples with 30 replacements, and the median and quartile values are plotted. **B**, **C**, Boxplot shows the distributions of Shannon diversity index (**B**) and the Simpson index (**C**) of gut bacteriome for two groups. **D**, PCoA analysis of Bray–Curtis distance based on the composition of gut bacteriome, revealing the separations between two groups. The location of samples (represented by nodes) in the first two principal coordinates is shown. Lines connect samples in the same group, and circles cover samples near the center of gravity for each group. **E**, Composition of gut bacteriome at the species level. **F**, Boxplot shows the representative differential gut bacterial species when compared between patient and control groups. **G**, **H**, Boxplot shows the Simpson index (**G**) and Shannon diversity index (**H**) of gut functional composition that significantly differs between patients and controls. **I**, PCoA analysis of Bray–Curtis distance based on the gut functional composition, revealing the separations between two groups. For boxplots, boxes represent the interquartile range between the first and third quartiles and median (internal line); whiskers denote the lowest and highest values within 1.5 times the range of the first and third quartiles, respectively; and nodes represent outliers beyond the whiskers. The significance level is calculated based on the Student’s *t*-test

Based on whole-metagenome shotgun sequencing, we obtained totaling 410.3 Gbp of high-quality non-human data (average 7.6 ± 2.4 Gbp per sample) from the fecal samples of 26 GA patients and 28 healthy individuals. Firstly, we mapped the sequencing reads of feces of all samples against the UHGG database [24] and obtained the gut prokaryotic profile (hereafter referred to as “gut bacteriome”) which contained a total of 5,728 bacterial and archaeal taxa, including 25 phyla, 34 classes, 81 orders, 216 families, 837 genera, and 4,535 species. Rarefaction analysis revealed that gut bacterial richness (estimated by the number of observed species) was approximately equal under the same sample size between patients and controls (Fig. 1A). However, both the Shannon diversity index and Simpson index were significantly lower in the gut bacteriome of GA patients compared with that of healthy controls (Fig. 1B-C), suggesting a reduced within-sample bacterial diversity under disease condition. We next undertook the PCoA and PERMANOVA analyses to further understand the differences in gut bacteriome between the two groups. A clear separation was revealed between the bacteriomes of the two groups, with the disease state explaining 16.6% of the bacteriome variances (PERMANOVA *p* < 0.001; Fig. 1D). These findings demonstrated considerable gut bacterial dysbiosis in GA patients.

At the phylum level, the gut bacteriome of GA patients had markedly higher levels of *Proteobacteria* (average abundance 20.8% in patients vs. 1.5% in controls, Wilcoxon rank-sum test *q* < 0.001) and *Actinobacteriota* (17.9% vs. 5.1%, *q* < 0.001) and lower level of *Bacteroidota* (2.2% vs. 27.2%, *q* < 0.001) and *Firmicutes_C* (2.2% vs. 5.8%, *q* < 0.001) compared with that of the healthy subjects (Table S1). At the genus level, 97 genera, including *Blautia_A*, *Escherichia*, *Streptococcus*, *Klebsiella*, and *Enterobacter* were enriched in GA patients compared with healthy controls, while 174 genera such as *Faecalibacterium*, *Prevotella, Bacteroides*, and *Agathobacter* were enriched in the healthy subjects (Table S2). In addition, we compared the gut bacteriome of GA and control subjects at the species level. 1,117 species were identified with significant differences in relative abundance between the two groups (Wilcoxon rank-sum test *q* < 0.01), while 366 of these species were enriched in GA patients and 751 of them were enriched in controls (Fig. 1E; Table S3). The representative GA-enriched species included *Escherichia coli_D GENOME144544*, *Bifidobacterium infantis GENOME095938*, *Blautia_A wexlerae GENOME096067*, *Klebsiella pneumoniae GENOME147598*, and *Escherichia fergusonii GENOME145983*, while the control-enriched species included *Faecalibacterium prausnitzii_G GENOME147678*, *Agathobacter rectalis GENOME143712*, *Bacteroides_A plebeius_A GENOME239725*, and *Faecalibacterium prausnitzii_K GENOME095967* (Fig. 1F; Fig. S1).

We profiled the functions of gut bacteriome in all fecal samples using the HUMAnN3 algorithm [25], representing a total of 479 MetaCyc pathways for comparison analysis between the GA patients and healthy controls. Diversity analysis at the pathway level uncovered notably higher Shannon and Simpson indexes in the gut functional composition of GA patients compared with that of controls (Wilcoxon rank-sum test *p* < 0.001; Fig. 1G-H). Consistent with the observation in the phylogenetic composition, the functional composition of two groups at the PCoA plot was also distinctly separated (PERMANOVA *R*^*2*^ = 35.6%, *p* = 0.001; Fig. 1I). Furthermore, we identified 195 of the 479 pathways that exhibited significant differences between the two cohorts (174 and 21 were enriched GA patients and healthy controls, respectively; Table S4). Representative GA-enriched pathways included acetylene degradation (MetaCyc ID: P161-PWY), gondoate biosynthesis (PWY-7663), octanoyl-[acyl-carrier protein] biosynthesis (PWY-7388), partial TCA cycle (PWY-5913), and inosine-5'-phosphate biosynthesis III (PWY-7234); while the representative control-enriched pathways included queuosine biosynthesis I (PWY-6700), L-histidine biosynthesis (HISTSYN-PWY), and pyrimidine deoxyribonucleosides salvage (PWY-7199).

### Diversity and phylogenetic comparisons of the gut mycobiome

**Fig. 2:**
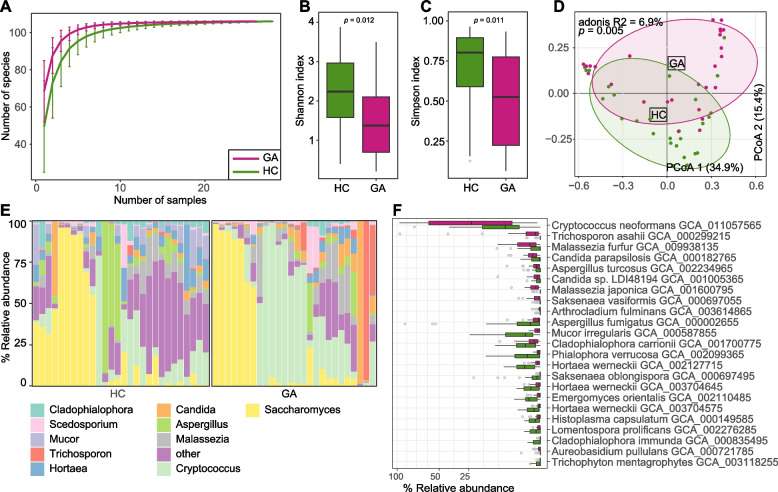
Difference in gut mycobiome between GA patients and healthy controls. **A**, Rarefaction curve analysis of the number of observed species in each group. The number of species in different groups is calculated based on a randomly selected specific number of samples with 30 replacements, and the median and quartile values are plotted. **B**, **C**, Boxplot shows the Shannon diversity index (**B**) and the Simpson index (**C**) of gut mycobiome that differ between two groups. **D**, PCoA analysis of Bray–Curtis distance based on the composition of gut mycobiome, revealing the separations between two groups. The location of samples (represented by nodes) in the first two principal coordinates is shown. Lines connect samples in the same group, and circles cover samples near the center of gravity for each group. **E**, Composition of gut mycobiome at the family level. **F**, Boxplot shows the GA-associated gut fungal species when compared between GA patients and healthy controls. For boxplots, boxes represent the interquartile range between the first and third quartiles and median (internal line); whiskers denote the lowest and highest values within 1.5 times the range of the first and third quartiles, respectively; and nodes represent outliers beyond the whiskers. The significance level is calculated based on the Student’s *t*-test

Next, we analyzed the gut fungal composition of all fecal samples based on the available fungal genome database in the NCBI database (see Methods). The composition of 106 fungal species (representing 1,503 fungal strains) was profiled and compared between the GA patients and healthy controls. Rarefaction analysis revealed that the number of observed species was approximately equal with the same sample size between the two groups (Fig. 2A). However, comparisons of fungal within-sample biodiversity revealed that the gut mycobiome was significantly different between the two groups in both the Shannon index and Simpson index (*p* < 0.05; Fig. 2B-C). Consistent with the observation in gut bacteriome, PCoA analysis of the gut mycobiome also showed a remarkable distinction between the patient and control groups (PERMANOVA *R*^*2*^ = 6.9%, *p* = 0.005; Fig. 2D). These findings underscored substantial gut mycobiome dysbiosis in patients with GA.

At the genus level, the gut mycobiome of GA patients was dominated by *Cryptococcus* (average abundance 33.3%), *Saccharomyces* (average abundance 12.8%), and *Malassezia* (average abundance 8.3%), while the healthy subjects were composed of *Saccharomyces* (average abundance 26.3%), *Cryptococcus* (average abundance 21.3%), and *Aspergillus* (average abundance 10.0%) (Fig. 2E). Of these, *Cryptococcus* significantly differed in abundance between the two cohorts (*q* = 0.04; Table S5). At the species level, 23 fungi differed in their relative abundances in the gut mycobiome between GA patients and healthy controls (Wilcoxon rank-sum test *q* < 0.01; Fig. 2F; Fig. S2). The GA-enriched species (*n* = 9) included several opportunistic pathogens such as *Cryptococcus neoformans* GCA_011057565, *Candida parapsilosis* GCA_000182765, and *Malassezia* spp., while the control-enriched species (*n* = 14) included several *Hortaea werneckii* subclades (GCA_002127715, GCA_003704645, and GCA_003704575), *Aspergillus fumigatus* GCA_000002655, among others.

### Gut virome cataloging and comparison between GA patients and healthy controls

**Fig. 3:**
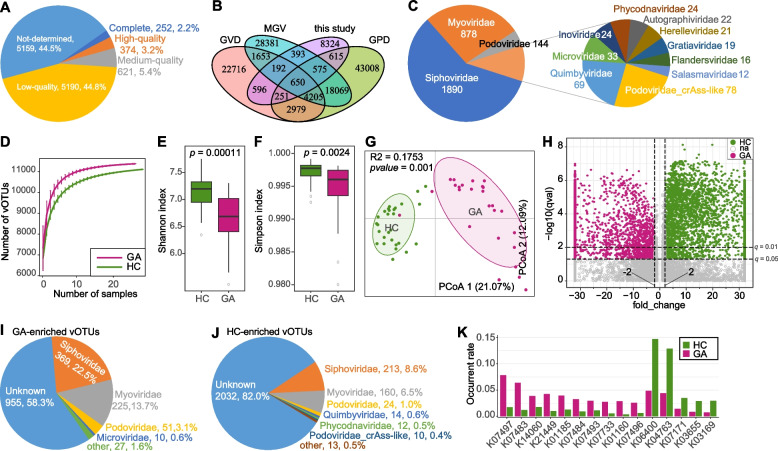
Characteristics of the gut virus catalog and gut virome. **A**, Pie plot shows the proportions of complete, high-quality, medium-quality, and low-quality vOTUs in the non-redundance virus catalog. **B**, Venn plot shows the overlap of the current virus catalog and the other three public gut virus catalogs. **C**, Pie plot shows the family-level taxonomic annotation of the virus catalog. **D**, Rarefaction curve analysis of the number of observed vOTUs on each group of samples. The number of species in different groups is calculated based on a randomly selected specific number of samples with 30 replacements, and the median and quartile values are plotted. **E**, **F**, Boxplot shows the Shannon diversity index (**E**) and the Simpson index (**F**) of gut virome that differ among two groups. Boxes represent the interquartile range between the first and third quartiles and median (internal line); whiskers denote the lowest and highest values within 1.5 times the range of the first and third quartiles, respectively; and nodes represent outliers beyond the whiskers. The significance level is calculated based on the Student’s *t*-test. **G**, PCoA analysis of Bray–Curtis distance based on the composition of gut virome, revealing the separations between two groups. The location of samples (represented by nodes) in the first two principal coordinates is shown. Lines connect samples in the same group, and circles cover samples near the center of gravity for each group. **H**, Volcano plot shows the fold change vs. q-values for all vOTUs. The X-axis shows the ratio of vOTU abundance in GA patients compared with that in healthy controls. The Y-axis shows the q-value (-log10 transformed) of a vOTU. The vOTUs that enriched in GA patients and healthy controls are shown in red and blue points, respectively. **I**, **J**, Pie plots show the taxonomic distribution of GA-enriched (**J**) and control-enriched (**K**) vOTUs. **K**, The occurrence rate of the KOs differed in frequency between the GA-enriched and ocntrol-enriched vOTUs

To unravel the gut viral signatures associated with GA, we assembled a total of 17,219 viral contigs (length ≥ 5,000 bp) from the metagenomic reads of all 54 fecal samples. These viral sequences enabled to cluster into 11,596 species-level vOTUs at 95% nucleotide similarity and 75% coverage. The length of this vOTU catalog ranged from 5,000 bp to 474,042 bp, with an average length of 15,119 bp and an N50 length of 22,509 bp. Based on the quality estimation using the CheckV algorithm [31], 2.2% of these vOTUs were evaluated as complete viral genomes, and 3.2% and 5.4% of them were high- and medium-quality viruses, respectively (Fig. 3A). Of note, only 28.2‬% (*n* = 3,272) of 11,596 vOTUs were shared with the currently available collections of the human gut virome including the Gut Virome Database [36], Gut Phage Database [37], and Metagenomic Gut Virus catalog [38] (Fig. 3B), which suggested a high novelty of our current viral catalog. Taxonomically, 28.1% of 11,596 vOTUs could be robustly assigned to a known viral family. Members of *Siphoviridae* (16.2%, *n* = 1,890) and *Myoviridae* (7.6%, *n* = 878) were dominated in the classified vOTUs, while other representative families included *Podoviridae*, *Quimbyviridae*, *Podoviridae_crAss-like*, *Autographiviridae*, *Herelleviridae*, *Inoviridae*, and *Phycodnaviridae* (Fig. 3C). Furthermore, 64.4% of the vOTUs could be assigned into one or more bacterial hosts based on their homology to genome sequences or CRISPR spacers of the prokaryotic genomes from the UHGG database. The most common identifiable hosts were members of *Firmicutes_A* (mainly *Lachnospiraceae* and *Ruminococcaceae*), *Actinobacteriota* (mainly *Coriobacteriaceae* and *Bifidobacteriaceae*), and *Bacteroidota* (mainly *Bacteroidaceae*, *Tannerellaceae*, and *Rikenellaceae*).

Rarefaction analysis showed that, the number of observed species was slightly higher in GA patients than that in healthy controls at the same number of individuals (Fig. 3D). However, both Shannon diversity and Simpson indexes revealed that the gut viral diversity is significantly decreased in GA patients compared with the controls (Student’s *t*-test *p* < 0.01, Fig. 3E-F). Consistently, PCoA analysis showed that the two groups are obviously different (PERMANOVA *R*^*2*^ = 17.5%, *p* = 0.001) in viral composition at the vOTU level (Fig. 3G).

Next, we compare the composition of gut virome between GA patients and healthy controls at the vOTU level. A total of 4,115 vOTUs had significantly differed in relative abundance between the two groups (Wilcoxon rank-sum test *q* < 0.01; Fig. 3H; Table S6); 1,637 of these were enriched in patients (GA-enriched) and 2,478 of these were decreased (control-enriched). The GA-enriched vOTUs encompassed 17 known viral families, primarily *Siphoviridae* (*n* = 369 vOTUs), *Myoviridae* (*n* = 225), *Podoviridae* (*n* = 51), and *Microviridae* (*n* = 10), whereas the control-enriched vOTUs spanned 13 families, chiefly featuring *Siphoviridae* (*n* = 213 vOTUs), *Myoviridae* (*n* = 160), and *Podoviridae* (*n* = 24) (Fig. 3I-J). In addition, we predicted a total of 76,265 protein-coding genes from the 4,115 differential vOTUs and annotated 27.7% of them based on the KEGG database [34]. These annotated genes were assigned into 2,914 KEGG orthologs (KOs) for further analyses. 42 KOs had significantly differed in occurrent frequency between the GA-enriched and control-enriched vOTUs (Wilcoxon rank-sum test *q* < 0.01; Fig. 3K; Table S7). 31 of these KOs were more frequent to be encoded in GA-enriched vOTUs, such as K01185 (lysozyme), K21449 (trimeric autotransporter adhesin), and K07451 (5-methylcytosine-specific restriction enzyme A); whereas 11 KOs include the K06400 (site-specific DNA recombinase), K04763 (integrase/recombinase), and K07171 (mRNA interferase), were enriched in the control-enriched vOTUs.

### Associations among gut bacteriome, mycobiome, and virome

**Fig. 4:**
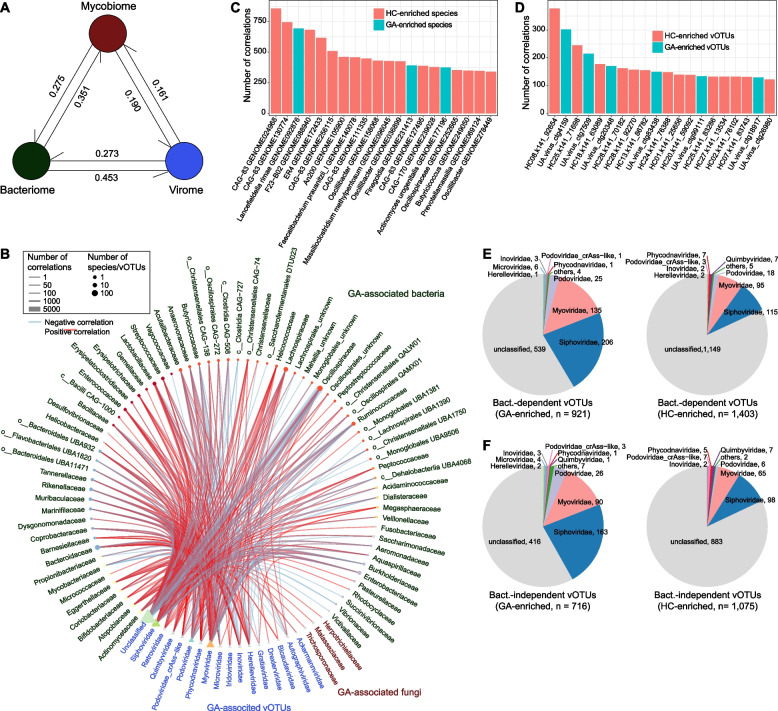
Interactions among gut bacteriome, mycobiome, and virome. **A**, The inter-omics effect sizes for the gut bacteriome, mycobiome, and virome. Numbers show the combined effect sizes between two datasets. **B**, Network showing the co-abundance correlations between gut bacteriome, mycobiome, and virome. All species and vOTUs are grouped at the family level. **C**, **D**, Barplots showing the number of top 20 gut bacterial species (**C**) and vOTUs (**D**) with the largest number of connections in the network. **E**, **F**, Pie plots showing the taxonomic distribution of bacterium-dependent (**E**) and bacterium-independent vOTUs (**F**)

To elucidate the intricate relationships between the various components of the gut microbiome, first, we performed a PERMANOVA-based analysis to estimate the effect size of variances between the gut bacteriome, mycobiome, and virome. This analysis found that the gut bacteriome contributed 35.1% and 45.3% of the overall variances of the gut mycobiome and virome, respectively (Fig. 4A). Meanwhile, the mycobiome and virome contributed 27.5% and 27.3%, respectively, of gut bacteriome variance. These findings suggested a considerable interaction between the gut bacteriome and mycobiome/virome. Inversely, the mycobiome and virome had relatively less influence, with effect sizes of 19.0% and 16.1% between each other.

We performed a correlation analysis between 1,117 GA-associated bacterial species, 23 GA-associated fungal species, and 4,115 GA-associated vOTUs. Using an absolute Spearman correlation coefficient threshold of 0.6, we generated a large co-abundance network between these multi-kingdom signatures (Fig. 4B). A majority of the correlations occurred between bacteria and viruses, whereas the correlations between fungi and bacteria/viruses were few. Several bacterial species, such as *Lancefieldella rimae GENOME092876* and *Faecalibacterium prausnitzii_I GENOME140078* had frequently connected to a large number of viruses, while some vOTUs were linked to the highest number of bacteria (Fig. 4C-D); these bacteria and viruses may play central roles in the network. Additionally, unlike these viruses (921 GA-enriched and 1,403 control-enriched vOTUs; Fig. 4E) that may depend on the gut bacteria to act in disease, the remaining vOTU signatures (716 GA-enriched and 1,075 control-enriched vOTUs; Fig. 4F) may act independently of the gut bacteriome, and their function needs to be further investigated.

### Classification of GA based on multi-kingdom signatures

**Fig. 5:**
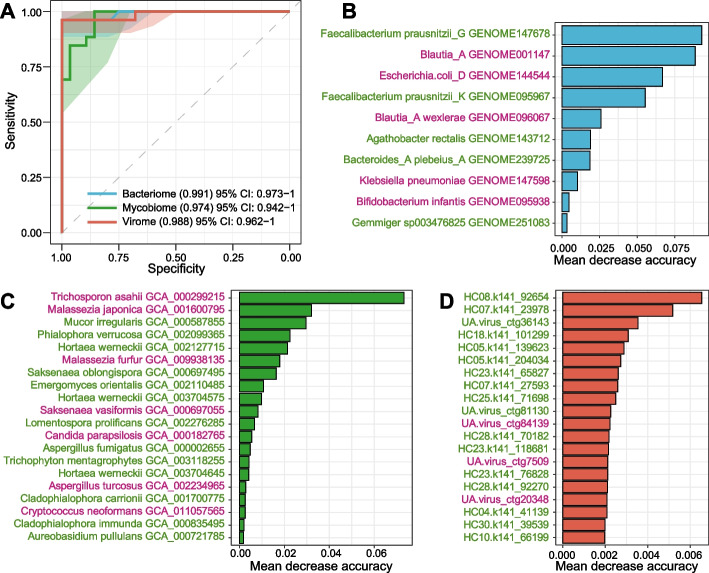
Classification of GA status by the compositions of gut multi-kingdom signatures. **A**, Receiver operating characteristic (ROC) analysis for classification of GA status using gut bacterial, fungal, and viral signatures. **B-D**, The 10 most important bacterial signatures (**B**), as well as the 20 most discriminant fungal (**C**) and viral signatures (**D**), in models aimed at classifying GA patients and healthy controls. The bar lengths indicate the importance of the variables and the label colors indicate the enriched trend of the microbial signatures (red: GA-enriched; green: control-enriched)

Finally, we evaluated the potential of the multi-kingdom signatures (including bacteriome, mycobiome, and virome signatures) of the fecal microbiome for the classification of GA status. Using the random forest model with leave-one-out cross-validation, we obtained the discriminatory powers of the area under the receiver operator characteristic curve (AUC) of 0.991, 0.974, and 0.988, respectively, for the models based on the gut bacterial, fungal, and viral signatures, respectively (Fig. 5A). Several bacteria, including *Faecalibacterium prausnitzii_G* GENOME147678, *Blautia_A* GENOME001147, *Escherichia coli_D* GENOME144544, and *Faecalibacterium prausnitzii_K* GENOME095967 features the highest important score for the discrimination of GA patients and healthy controls (Fig. 5B). Also, the gut fungal and viral signatures with the highest scores were listed in Fig. 5C-D; the roles of these signatures in GA and related diseases deserve further exploration.

## Discussion

GA is classified as a crystal-related arthropathy caused by the deposition of monosodium urate crystals due to hyperuricemia [2]. The relationship between immune diseases and gut microbiota in GA patients remains inadequately understood. In this study, we conducted whole-metagenome sequencing of fecal samples from 26 GA patients and 28 healthy controls to investigate microbial compositions in these two cohorts. Our comparative analysis revealed that GA patients exhibited significantly reduced diversity and differences in the abundance of 1,117 bacterial, 106 fungal, and 4,115 viral species. This distinctive gut microbial signature may provide valuable insights into the understanding of GA and related immunological diseases.

We observed a noteworthy decrease in bacterial diversity among GA patients, a shared characteristic in various immunological diseases, indicating the emergence of gut microbiota dysbiosis. Notably, the disease state contributed to 16.6% of the variance in the gut bacteriome, a proportion significantly larger than observed in previous studies of RA or OA [6, 39], suggesting that GA patients may experience more pronounced shifts in their microbiota. Specifically, we identified an enrichment of *Blautia_A* (primarily *Blautia_A wexlerae*), *Streptococcus*, and *Enterobacteriaceae* (including *Escherichia*, *Klebsiella*, and *Enterobacter* spp.) in the gut bacteriome of GA patients. *Blautia wexlerae*, a major producer of butyrate, has shown promise in improving insulin resistance and reducing fat accumulation in animal experiments [40]. However, *Blautia wexlerae* has also been associated with diabetes [41] and was depleted in obese and insulin-resistant children and inversely associated with inflammatory markers in feces [42], suggesting that the role of this bacterium in various diseases warrants further investigation. *Streptococcus* is considered an opportunistic pathogen and has been linked to rheumatic diseases, including rheumatic fever and RA [43]. *Streptococcus* was found to be more prevalent in hyperuricemia (HUA) children and in patients with gout [44]. Similarly, *Enterobacteriaceae* overgrowth in the gut also indicates harmful functions, a phenomenon observed in various diseases, including RA, SLE, and IBD [45–47]. These findings suggest potential roles for *Streptococcus* and *Enterobacteriaceae* in promoting the development of GA. Additionally, we observed an increase in *Bifidobacterium infantis* in GA patients. *B. infantis* is a probiotic with potential immunomodulatory effects, and its role in the gut microbiota of GA patients warrants further investigation. Conversely, GA patients exhibited a deficiency in some crucial bacteria, such as *Prevotella*, which metabolizes plant polysaccharides and produces SCFAs [48], and *Bacteroides*, which metabolizes animal polysaccharides and produces vitamins [49]. Typical SCFA-producing bacteria in the human gut [50], *Faecalibacterium prausnitzii*, was also notably decreased in GA patients. *F. prausnitzii* possesses anti-inflammatory properties and promotes intestinal health through butyrate production [51]. These bacteria hold promise as indicators of gut health. In summary, our findings may contribute to our understanding and interpretation of the disease's etiology.

The gut mycobiome of GA patients revealed an increased relative abundance of pathogenic *Cryptococcus* and decreased potential commensal bacteria such as *Saccharomyces*. *Cryptococcus* is associated with infectious diseases, causing conditions such as pulmonary cryptococcal disease and meningitis with various complications [52]. *Candida albicans*, an opportunistic pathogen, also saw increase in GA patients and is known to interact with the local gut microbiota, affecting the severity of infections [53]. *Saccharomyces*, a non-pathogenic selective probiotic, has been used in commercial biotic probiotic food production [54] and associated with increased *Bacteroides* proportion and decreased abundances of *Firmicutes* and *Proteobacteria*, known to prevent inflammation and promote immune function [55]. These findings emphasize gut mycobiome dysbiosis in GA patients, necessitating further investigation into its role in the disease. The increased abundance of pathogenic *Cryptococcus* and *Candida*, coupled with the decreased presence of probiotic *Saccharomyces*, indicates an imbalance in the gut microbiota, potentially contributing to GA development.

The gut virome in GA patients exhibited a significant reduction in viral diversity, a pattern also observed in other arthritis patients, including RA and OA [6, 16]. At the vOTU level, *Microviridae* viruses were more abundant in GA patients, while certain viruses belonging to *Quimbyviridae* and *crAss-like* were decreased. *Microviridae* encompasses a family of small ssDNA phages that lack a tail structure and infect Gram-negative bacteria [56]. However, recent studies have shown a depletion of *Microviridae* in patients with IBD, IBS, Crohn's disease, and coronary heart disease [57–59]. *Quimbioviridae* is a highly abundant and widely prevalent viral family in the human gut, considered an obligate lytic phage, with some *Quimbioviridae* phages producing retroelements (DGRs) [60]. *crAss-like* phages are the most abundant viruses in the healthy human gut but were significantly decreased in patients with RA, SLE, and IBD [61]. We also established a broad connection between viruses and bacteria, with 4,115 GA-related viruses associated with bacteria, and some viruses acting independently of bacteria. The gut viral diversity of GA patients requires further study, particularly of unknown viruses and their function.

Our study has limitations, including the relatively small sample size, and potential population factors such as sex and age were not entirely excluded. Large-scale studies are warranted in the future. Moreover, we were unable to eliminate the potential influence of medication on the gut microbiota in GA. Lastly, our results are based on a correlation study of the gut microbiota in GA patients, and no subsequent mechanistic validation, such as in animal experiments, was conducted. However, our study provides a foundational basis for future experiments.

## Conclusions

Overall, we have systematically characterized the gut bacteriome, mycobiome, and virome in GA patients for the first time, employing whole-metagenome shotgun sequencing of their fecal samples. Our findings reveal a profound reshaping of the gut microbiome in GA patients when compared to their healthy counterparts. This transformation is underscored by the identification of a diverse set of 1,117 differentially abundant bacterial species, 23 fungal species, and 4,115 vOTUs. Furthermore, by delving into functional analysis and exploring the interconnected signatures of GA-associated gut microbiota across multiple microbial kingdoms, we have shed light on potential links between gut microbiota and GA. Our study lays the foundation for future investigations aimed at uncovering the mechanistic underpinnings of these microbial alterations in GA. This research not only holds promise for the development of innovative therapeutic strategies for GA but also opens doors to explore the role of the microbiome in similar inflammatory conditions.

### Supplementary Information

**Additional file 1: Table S1.** Comparison of the gut bacteriome between GA patients and healthy controls at the phylum level.** Table S2.** Comparison of the gut bacteriome between GA patients and healthy controls at the genus level.** Table S3.** Comparison of the gut bacteriome between GA patients and healthy controls at the species level.** Table S4.** Comparison of the gut functional composition between GA patients and healthy conrols. **Table S5.** Comparison of the gut mycobiome between GA patients and healthy controls at the genus level. **Table S6.** Comparison of the gut virome between HC and GA subjects at the vOTUs level. Only vOTUs that differed in abundance between two groups are shown. **Table S7.** Detailed information of 42 KOs that differed in frequency between the GA-enriched and HC-enriched vOTUs.

**Additional file 2: Figure S1. **Heatmap showing the distribution of relative abundances of representative bacterial species that enriched in GA patients or healthy controls. **Figure S2. **Heatmap showing the distribution of relative abundances of 23 fungal species that enriched in GA patients or healthy controls.

## Data Availability

The raw metagenomic sequencing dataset for this study has been deposited in the European Nucleotide Archive (ENA) at EMBL-EBI under accession number PRJEB58696 (https://www.ebi.ac.uk/ena/data/view/PRJEB58696). The authors declare that all other data supporting the findings of the study are available in the paper and supplementary materials, or from the corresponding authors upon request.

## References

[1] Dalbeth N, Choi HK, Joosten LAB, Khanna PP, Matsuo H, Perez-Ruiz F, Stamp LK (2019). Gout. Nat Rev Dis Primers.

[2] Galozzi P, Bindoli S, Doria A, Oliviero F, Sfriso P. Autoinflammatory Features in Gouty Arthritis. J Clin Med. 2021;10(9):1880. 10.3390/jcm10091880 PMC8123608 33926105

[3] Dalbeth N, Merriman TR, Stamp LK (2016). Gout. Lancet.

[4] Punzi L, Scanu A, Galozzi P, Luisetto R, Spinella P, Scirè CA, Oliviero F (2020). One year in review 2020: gout. Clin Exp Rheumatol.

[5] Li M, Wang F (2021). Role of Intestinal Microbiota on Gut Homeostasis and Rheumatoid Arthritis. J Immunol Res.

[6] Chen C, Zhang Y, Yao X, Li S, Wang G, Huang Y, Yang Y, Zhang A, Liu C, Zhu D (2023). Characterizations of the Gut Bacteriome, Mycobiome, and Virome in Patients with Osteoarthritis. Microbiol Spectrum.

[7] Weingarden AR, Vaughn BP (2017). Intestinal microbiota, fecal microbiota transplantation, and inflammatory bowel disease. Gut microbes.

[8] Pan Q, Guo F, Huang Y, Li A, Chen S, Chen J, Liu HF, Pan Q (2021). Gut Microbiota Dysbiosis in Systemic Lupus Erythematosus: Novel Insights into Mechanisms and Promising Therapeutic Strategies. Front Immunol.

[9] Chen C, Yan Q, Yao X, Li S, Lv Q, Wang G, et al. Alterations of the gut virome in patients with systemic lupus erythematosus. Front Immunol. 2023;13:1050895. 10.3389/fimmu.2022.1050895 PMC9874095 36713446

[10] Zaky A, Glastras SJ, Wong MYW, Pollock CA, Saad S. The Role of the Gut Microbiome in Diabetes and Obesity-Related Kidney Disease. Int J Mol Sci. 2021;22(17):9641. 10.3390/ijms22179641 PMC8431784 34502562

[11] Inamo J (2021). Non-causal association of gut microbiome on the risk of rheumatoid arthritis: a Mendelian randomisation study. Ann Rheum Dis.

[12] Rogier R, Ederveen THA, Boekhorst J, Wopereis H, Scher JU, Manasson J, Frambach S, Knol J, Garssen J, van der Kraan PM (2017). Aberrant intestinal microbiota due to IL-1 receptor antagonist deficiency promotes IL-17- and TLR4-dependent arthritis. Microbiome.

[13] Chen BD, Jia XM, Xu JY, Zhao LD, Ji JY, Wu BX, Ma Y, Li H, Zuo XX, Pan WY (2021). An Autoimmunogenic and Proinflammatory Profile Defined by the Gut Microbiota of Patients With Untreated Systemic Lupus Erythematosus. Arthritis Rheumatol (Hoboken, NJ).

[14] Azzouz D, Omarbekova A, Heguy A, Schwudke D, Gisch N, Rovin BH, Caricchio R, Buyon JP, Alekseyenko AV, Silverman GJ (2019). Lupus nephritis is linked to disease-activity associated expansions and immunity to a gut commensal. Ann Rheum Dis.

[15] Sun X, Wang Y, Li X, Wang M, Dong J, Tang W, Lei Z, Guo Y, Li M, Li Y (2022). Alterations of gut fungal microbiota in patients with rheumatoid arthritis. PeerJ.

[16] Guo R, Li S, Zhang Y, Zhang Y, Wang G, Ullah H, et al. Dysbiotic Oral and Gut Viromes in Untreated and Treated Rheumatoid Arthritis Patients. Microbiol Spectrum. 2022;10(5):e0034822. 10.1128/spectrum.00348-22 PMC9603985 36040159

[17] Shin NR, Whon TW, Bae JW (2015). Proteobacteria: microbial signature of dysbiosis in gut microbiota. Trends Biotechnol.

[18] Méndez-Salazar EO, Vázquez-Mellado J, Casimiro-Soriguer CS, Dopazo J, Çubuk C, Zamudio-Cuevas Y, Francisco-Balderas A, Martínez-Flores K, Fernández-Torres J, Lozada-Pérez C (2021). Taxonomic variations in the gut microbiome of gout patients with and without tophi might have a functional impact on urate metabolism. Mol Med (Cambridge, Mass).

[19] Lin X, Shao T, Huang L, Wen X, Wang M, Wen C, He Z (2020). Simiao Decoction Alleviates Gouty Arthritis by Modulating Proinflammatory Cytokines and the Gut Ecosystem. Front Pharmacol.

[20] Park HK, Lee SJ (2022). Treatment of gouty arthritis is associated with restoring the gut microbiota and promoting the production of short-chain fatty acids. Arthritis Res Ther.

[21] Neogi T, Jansen TL, Dalbeth N, Fransen J, Schumacher HR, Berendsen D, Brown M, Choi H, Edwards NL, Janssens HJ (2015). 2015 Gout classification criteria: an American College of Rheumatology/European League Against Rheumatism collaborative initiative. Ann Rheum Dis.

[22] Chen S, Zhou Y, Chen Y, Gu J (2018). fastp: an ultra-fast all-in-one FASTQ preprocessor. Bioinformatics.

[23] Langmead B, Salzberg SL (2012). Fast gapped-read alignment with Bowtie 2. Nat Methods.

[24] Almeida A, Nayfach S, Boland M, Strozzi F, Beracochea M, Shi ZJ, Pollard KS, Sakharova E, Parks DH, Hugenholtz P, et al. A unified catalog of 204,938 reference genomes from the human gut microbiome. Nat Biotechnol. 2021;39(1):105–14. 10.1038/s41587-020-0603-3 PMC7801254 32690973

[25] Beghini F, McIver LJ, Blanco-Miguez A, Dubois L, Asnicar F, Maharjan S, Mailyan A, Manghi P, Scholz M, Thomas AM, et al. Integrating taxonomic, functional, and strain-level profiling of diverse microbial communities with bioBakery 3. Elife. 2021;10:e65088. 10.7554/eLife.65088 PMC8096432 33944776

[26] Li D, Liu CM, Luo R, Sadakane K, Lam TW (2015). MEGAHIT: an ultra-fast single-node solution for large and complex metagenomics assembly via succinct de Bruijn graph. Bioinformatics.

[27] Yan Q, Wang Y, Chen X, Jin H, Wang G, Guan K, Zhang Y, Zhang P, Ayaz T, Liang Y (2021). Characterization of the gut DNA and RNA Viromes in a Cohort of Chinese Residents and Visiting Pakistanis. Virus Evol..

[28] Li S, Guo R, Zhang Y, Li P, Chen F, Wang X, et al. A catalogue of 48,425 nonredundant viruses from oral metagenomes expands the horizon of the human oral virome. iScience. 2022;25(6):104418. 10.1016/j.isci.2022.104418 PMC9160773 35663034

[29] Li S, Yan Q, Wang G, Zhang Y, Guo R, Zhang P, Lv Q, Chen F, Zhiming LI, Meng J, et al. Cataloguing and profiling of the gut virome in Chinese populations uncover extensive viral signatures across common diseases. bioRxiv. 2022:2022-12.

[30] Wang G, Li S, Yan Q, Guo R, Zhang Y, Chen F, Tian X, Lv Q, Jin H, Ma X, et al. Optimization and evaluation of viral metagenomic amplification and sequencing procedures toward a genome-level resolution of the human fecal DNA virome. J Adv Res. 2023;48:75–86. 10.1016/j.jare.2022.08.011 PMC10248800 35995413

[31] Nayfach S, Camargo AP, Schulz F, Eloe-Fadrosh E, Roux S, Kyrpides NC. CheckV assesses the quality and completeness of metagenome-assembled viral genomes. Nat Biotechnol. 2021;39(5):578–85. 10.1038/s41587-020-00774-7 PMC8116208 33349699

[32] Mihara T, Nishimura Y, Shimizu Y, Nishiyama H, Yoshikawa G, Uehara H, Hingamp P, Goto S, Ogata H (2016). Linking Virus Genomes with Host Taxonomy. Viruses.

[33] Bin Jang H, Bolduc B, Zablocki O, Kuhn JH, Roux S, Adriaenssens EM, Brister JR, Kropinski AM, Krupovic M, Lavigne R (2019). Taxonomic assignment of uncultivated prokaryotic virus genomes is enabled by gene-sharing networks. Nat Biotechnol.

[34] Kanehisa M, Furumichi M, Tanabe M, Sato Y, Morishima K (2017). KEGG: new perspectives on genomes, pathways, diseases and drugs. Nucleic Acids Res.

[35] Su G, Morris JH, Demchak B, Bader GD (2014). Biological network exploration with Cytoscape 3. Curr Protoc Bioinformatics..

[36] Gregory AC, Zablocki O, Zayed AA, Howell A, Bolduc B, Sullivan MB (2020). The Gut Virome Database Reveals Age-Dependent Patterns of Virome Diversity in the Human Gut. Cell Host Microbe..

[37] Camarillo-Guerrero LF, Almeida A, Rangel-Pineros G, Finn RD, Lawley TD (2021). Massive expansion of human gut bacteriophage diversity. Cell.

[38] Nayfach S, Paez-Espino D, Call L, Low SJ, Sberro H, Ivanova NN, Proal AD, Fischbach MA, Bhatt AS, Hugenholtz P (2021). Metagenomic compendium of 189,680 DNA viruses from the human gut microbiome. Nat Microbiol.

[39] Zhang X, Zhang D, Jia H, Feng Q, Wang D, Liang D, Wu X, Li J, Tang L, Li Y (2015). The oral and gut microbiomes are perturbed in rheumatoid arthritis and partly normalized after treatment. Nat Med.

[40] Liu X, Mao B, Gu J, Wu J, Cui S, Wang G, Zhao J, Zhang H, Chen W (2021). Blautia-a new functional genus with potential probiotic properties?. Gut Microbes.

[41] Yang Y, Liu S, Wang Y, Wang Z, Ding W, Sun X, He K, Feng Q, Zhang X (2020). Changes of saliva microbiota in the onset and after the treatment of diabetes in patients with periodontitis. Aging.

[42] Benítez-Páez A, Gómez Del Pugar EM, López-Almela I, Moya-Pérez Á, Codoñer-Franch P, Sanz Y. Depletion of Blautia Species in the Microbiota of Obese Children Relates to Intestinal Inflammation and Metabolic Phenotype Worsening. mSystems. 2020;5(2):e00857–19. 10.1128/mSystems.00857-19 PMC7093825 32209719

[43] Konig MF (2020). The microbiome in autoimmune rheumatic disease. Best Pract Res Clin Rheumatol.

[44] Yuan X, Chen R, Zhang Y, Lin X, Yang X (2022). Altered Gut Microbiota in Children With Hyperuricemia. Front Endocrinol.

[45] Chiang HI, Li JR, Liu CC, Liu PY, Chen HH, Chen YM, et al. An Association of Gut Microbiota with Different Phenotypes in Chinese Patients with Rheumatoid Arthritis. J Clin Med. 2019;8(11):1770. 10.3390/jcm8111770 PMC6912313 31652955

[46] Xiang S, Qu Y, Qian S, Wang R, Wang Y, Jin Y, et al. Association between systemic lupus erythematosus and disruption of gut microbiota: a meta-analysis. Lupus Sci Med. 2022;9(1):e000599. 10.1136/lupus-2021-000599 PMC8961174 35346981

[47] Zuo T, Ng SC (2018). The Gut Microbiota in the Pathogenesis and Therapeutics of Inflammatory Bowel Disease. Front Microbiol.

[48] Connolly ML, Lovegrove JA, Tuohy KM (2010). In vitro evaluation of the microbiota modulation abilities of different sized whole oat grain flakes. Anaerobe.

[49] Zafar H, Saier MH (2021). Gut Bacteroides species in health and disease. Gut Microbes.

[50] Zhou Y, Xu H, Xu J, Guo X, Zhao H, Chen Y, Zhou Y, Nie Y (2021). F. prausnitzii and its supernatant increase SCFAs-producing bacteria to restore gut dysbiosis in TNBS-induced colitis. AMB Express..

[51] Guo Z, Zhang J, Wang Z, Ang KY, Huang S, Hou Q, Su X, Qiao J, Zheng Y, Wang L (2016). Intestinal Microbiota Distinguish Gout Patients from Healthy Humans. Sci Rep.

[52] Francisco EC, de Jong AW, Hagen F (2021). Cryptococcosis and Cryptococcus. Mycopathologia.

[53] d'Enfert C, Kaune AK, Alaban LR, Chakraborty S, Cole N, Delavy M, Kosmala D, Marsaux B, Fróis-Martins R, Morelli M, et al. The impact of the Fungus-Host-Microbiota interplay upon Candida albicans infections: current knowledge and new perspectives. FEMS Microbiol Rev. 2021;45(3):fuaa060. 10.1093/femsre/fuaa060 PMC8100220 33232448

[54] Yu L, Zhao XK, Cheng ML, Yang GZ, Wang B, Liu HJ, Hu YX, Zhu LL, Zhang S, Xiao ZW (2017). Saccharomyces boulardii Administration Changes Gut Microbiota and Attenuates D-Galactosamine-Induced Liver Injury. Sci Rep.

[55] Li M, Zhu L, Xie A, Yuan J (2015). Oral administration of Saccharomyces boulardii ameliorates carbon tetrachloride-induced liver fibrosis in rats via reducing intestinal permeability and modulating gut microbial composition. Inflammation.

[56] Shkoporov AN, Clooney AG, Sutton TDS, Ryan FJ, Daly KM, Nolan JA, McDonnell SA, Khokhlova EV, Draper LA, Forde A (2019). The Human Gut Virome Is Highly Diverse, Stable, and Individual Specific. Cell Host Microbe.

[57] Duan Y, Young R, Schnabl B (2022). Bacteriophages and their potential for treatment of gastrointestinal diseases. Nat Rev Gastroenterol Hepatol.

[58] Mihindukulasuriya KA, Mars RAT, Johnson AJ, Ward T, Priya S, Lekatz HR, Kalari KR, Droit L, Zheng T, Blekhman R (2021). Multi-Omics Analyses Show Disease, Diet, and Transcriptome Interactions With the Virome. Gastroenterology.

[59] Norman JM, Handley SA, Baldridge MT, Droit L, Liu CY, Keller BC, Kambal A, Monaco CL, Zhao G, Fleshner P (2015). Disease-specific alterations in the enteric virome in inflammatory bowel disease. Cell.

[60] Benler S, Yutin N, Antipov D, Rayko M, Shmakov S, Gussow AB, Pevzner P, Koonin EV (2021). Thousands of previously unknown phages discovered in whole-community human gut metagenomes. Microbiome.

[61] Tomofuji Y, Kishikawa T, Maeda Y, Ogawa K, Nii T, Okuno T, Oguro-Igashira E, Kinoshita M, Yamamoto K, Sonehara K (2022). Whole gut virome analysis of 476 Japanese revealed a link between phage and autoimmune disease. Ann Rheum Dis.

