# Supplementary material for: Characterizations of the multi-kingdom gut microbiota in Chinese patients with gouty arthritis
Source: BMC Microbiol. 2023 Nov 24;23:363. doi: 10.1186/s12866-023-03097-0 (PMC10668524; doi:10.1186/s12866-023-03097-0)
Supplement: Supplementary file 2 — Additional file 2: Figure S1. Heatmap showing the distribution of relative abundances of representative bacterial species that enriched in GA patients or healthy controls. Figure S2. Heatmap showing the distribution of relative abundances of 23 fungal species that enriched in GA patients or healthy controls. [file 12866_2023_3097_MOESM2_ESM.docx]

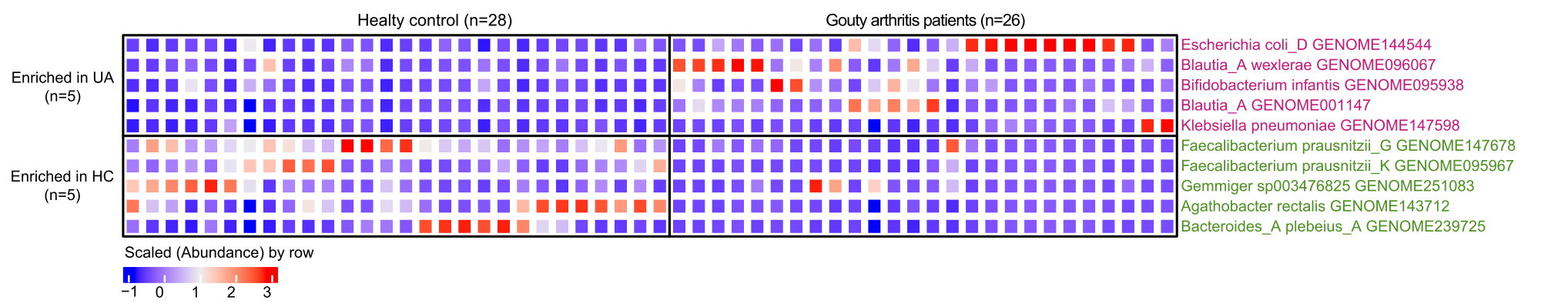


**Figure S1.** Heatmap showing the distribution of relative abundances of representative bacterial species that enriched in GA patients or healthy controls.


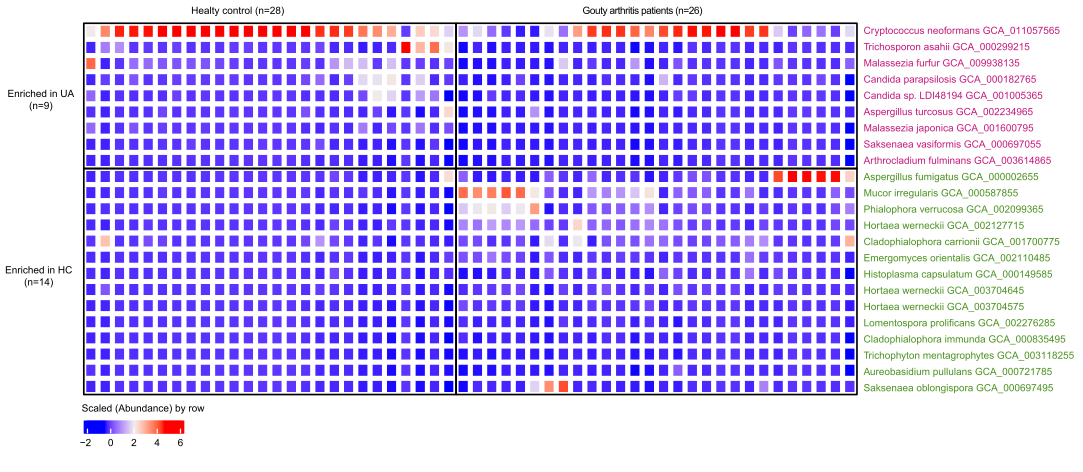


**Figure S2.** Heatmap showing the distribution of relative abundances of 23 fungal species that enriched in GA patients or healthy controls.
